# Quantifying Intermodal Distraction by Emotion During Math Performance: An Electrophysiological Approach

**DOI:** 10.3389/fpsyg.2019.00439

**Published:** 2019-03-12

**Authors:** Sabine Heim, Andreas Keil

**Affiliations:** ^1^ Infancy Studies Laboratory, Center for Molecular and Behavioral Neuroscience, Rutgers University-Newark, Newark, NJ, United States; ^2^ Psychophysiology Laboratory, Department of Psychology, Center for the Study of Emotion and Attention, University of Florida, Gainesville, FL, United States

**Keywords:** EEG, steady-state visual evoked potential, arithmetic, visual attention, auditory distraction, emotional arousal, temporal competition

## Abstract

Emotionally engaging stimuli are powerful competitors for limited attention capacity. In the cognitive neuroscience laboratory, the presence of task-irrelevant emotionally arousing visual distractors prompts decreased performance and attenuated brain responses measured in concurrent visual tasks. The extent to which distraction effects occur across different sensory modalities is not yet established, however. Here, we examined the extent and time course of competition between a naturalistic distractor sound and a visual task stimulus, using dense-array electroencephalography (EEG) recordings from 20 college students. Steady-state visual evoked potentials (ssVEPs) were quantified from EEG, elicited by periodically flickering vignettes displaying basic arithmetic problems – the participants’ primary task. Concurrently, low-arousing and high-arousing sounds were presented, as well as auditory pink noise, used as a control. Capitalizing on the temporal dynamics of the ssVEP signal allowed us to study intermodal interference of the sounds with the processing of the visual math problems. We observed that high-arousing sounds were associated with diminished visuocortical responses and poor performance, compared to low-arousing sounds and pink noise, suggesting that emotional distraction acts across modalities. We discuss the role of sensory cortices in emotional distraction along with implications for translational research in educational neuroscience.

## Introduction

The rapid and effective analysis of sensory information relevant to survival is critical to adaptive behavior. In line with this notion, the sensory representation of emotionally engaging stimuli, for instance, cues signaling threat or reward, is enhanced, compared to cues lacking behavioral or biological relevance (Bradley et al., 2012). This phenomenon has been referred to as “motivated attention,” which is directed to emotionally arousing (appetitive or aversive) stimuli without explicit instruction (Lang et al., 1997). Empirically, an impressive number of studies have observed heightened activity in sensory cortices during motivated attention, using electrophysiology (Cuthbert et al., 2000; Keil et al., 2002; Liu et al., 2012) as well as functional imaging techniques (Bradley et al., 2003; Sabatinelli et al., 2011). Contemporary audiovisual media, such as the World Wide Web, computer games, and television, rely heavily on capturing attention by means of changing motivationally salient stimuli such as violent or erotic scenes (Heim et al., 2013a; Iordan and Dolcos, 2015). Although the attention-grabbing potential of affective stimuli has been well established (Bradley et al., 2012; Deweese et al., 2014), the consequences of affective attention capture on the performance in competing or concurrent tasks are unclear. Two central competing hypotheses have been discussed in the literature.

One perspective assumes that the presence of emotionally arousing stimuli heightens attentional resources and therefore facilitates processing of concurrent stimuli. We refer to this hypothesis as the *supplementation hypothesis* (Bradley et al., 2012). It is exemplified in magazines, television commercials, websites, etc., in which the central information is presented together with emotionally arousing content (e.g., cartoons, erotica, or even mutilation and threat) to “attract attention.” This view predicts enhanced processing of concurrent information when emotional stimuli are present. A second perspective holds that, because affective stimuli tend to capture and hold attentional resources, they are powerful competitors and thus will act as distractors with respect to concurrent cognitive tasks. This *distraction hypothesis* predicts poorer processing of task stimuli when accompanied by emotionally engaging stimuli (Bradley et al., 2012).

Previous research has not only provided some support for the competition/distraction hypothesis (Ihssen et al., 2007; Heim et al., 2013a; Deweese et al., 2014) but also identified situations in which emotionally engaging cues benefit task processing (Leppänen et al., 2003; Thigpen et al., 2016). Although no consensus has been reached, some of the extant research can be taken to suggest that distraction is most likely in situations where the task stimulus and the distractor draw on the same pool of resources, for example, when both are visually presented in the same location in space, at the same time (Müller et al., 2008). One key aspect related to this problem is the extent to which there is supramodal competition or facilitation between task stimuli and emotional distractors; that is, do distractors and task stimuli compete even when presented in different (e.g., visual and auditory) modalities? This question is of both theoretical and practical importance, with implications for topics, such as web design, and for the production of multimedia learning material.

The goal of the present research is to determine whether emotionally engaging sound distractors benefit or attenuate processing of a concurrent visual arithmetic task, using electrocortical measures with high temporal fidelity. Several tasks have been implemented to explore competitive interactions between multiple stimuli in selective attention research. Specifically, detection and discrimination paradigms have been proven as a method of choice. In the current study, we were interested in potential translation of laboratory-based research on intermodal competition effects to academic tasks. Therefore, we adapted a simple mathematics test (Woodcock et al., 2001) to serve as the primary task for use with the frequency tagging method (see below). This approach has potential for understanding the effects of modern media, produced for recreation and entertainment, on concurrent processing. It may also inform the design of materials for training and education settings. We use electrocortical steady-state visual evoked potentials (ssVEPs; Regan and Spekreijse, 1986; Norcia et al., 2015; Wieser et al., 2016). Frequency-tagged ssVEPs are a special case of oscillatory brain responses, arising when a visual stimulus is rapidly and periodically modulated in luminance or contrast. These responses can then be readily separated in the frequency domain from an auditory response co-occurring at the same time but lacking the regular modulation driving the ssVEP. The ssVEP methodology has two key properties that make it a valuable tool for investigations of motivated attention dynamics as defined above: First, the amplitude of the ssVEP represents a continuous index of attentional resource allocation to visual stimuli (e.g., Müller et al., 2008). A second important advantage of ssVEPs is that attention allocation to a specific stimulus can be measured even when this stimulus is embedded in a complex intermodal array (Norcia et al., 2015).

A key conceptual issue of the present work is the extent to which emotional distraction may act across modalities. Previous research has illustrated the limitation of attending information streams in two modalities simultaneously. There is abundant evidence of dramatic performance costs when participants are asked to attend to information in more than one modality, e.g., driving a car while concurrently judging spoken sentences (Just et al., 2008), especially when the multimodal task is difficult (Jolicoeur, 1999). At the neural level, symmetrical cost effects between visual and auditory processing have been reported, in which heightened visuocortical activity during attentive processing was at the cost of auditory sensory processing and vice versa (Johnson and Zatorre, 2005, 2006).

In the study described here, we flickered a stream of visually presented math problems at a rate of 10 Hz, driving the ssVEP signal in the visual cortex of college students at the same temporal frequency. This frequency is in the alpha (8–13 Hz) range, where high-amplitude oscillations dominate the spontaneous EEG. Previous methodological work has demonstrated that with sufficient trial averaging prior to time-frequency analysis, the 10 Hz ssVEP provides excellent signal-to-noise as well as variation with experimental manipulations that parallel those seen in non-alpha driving frequencies. Concurrently, we delivered auditory distractors varying in emotional content, namely low-arousing and high-arousing sounds, as well as a pink noise control. If the intermodal distraction hypothesis is supported, then the behavioral accuracy and ssVEP signal evoked by the math-problem stream will be attenuated in the presence of emotionally engaging (i.e., high-arousing) auditory distractors. By contrast, if the supplementation hypothesis holds, then accuracy and the ssVEP are expected to be heightened during high-arousing sound distraction.

## Materials and Methods

### Participants

Twenty undergraduate college students at the University of Florida (five males; mean age 18.45 ± 1.36 years) volunteered in the present research. Participants were right-handed according to the Edinburgh Handedness Inventory (laterality quotient ≥90; Oldfield, 1971), had normal or corrected-to-normal vision, and no history of photic epilepsy. Informed written consent was obtained from all participants prior to inclusion into the study. Students received course credit for their participation. All procedures conformed to the Declaration of Helsinki and were approved by the University of Florida’s Institutional Review Board.

### Stimuli and Task Paradigm: The Intermodal Distraction Task

**Figure 1:**
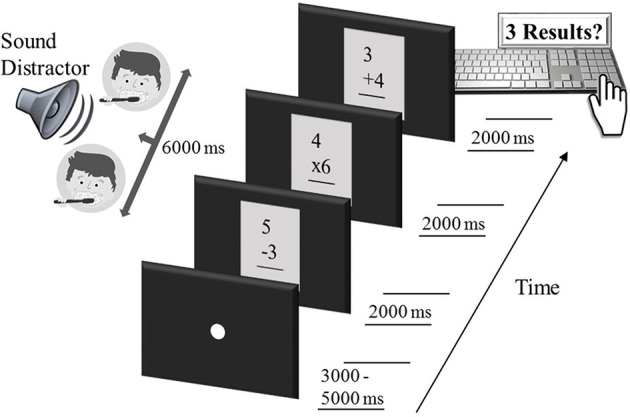
Sequence of an example trial in the intermodal distraction task. Each trial started with a white fixation dot, lasting 3,000–5,000 ms. Subsequently, three single-digit arithmetic problems were shown, each for a duration of 2000 ms, while an auditory distractor (here, brush teeth, falling into the category of low-arousing sounds) was delivered free-field to the participant for the entire period of 6,000 ms. Participants then responded by typing the three calculation results on the number pad of a computer keyboard. After another variable fixation period of 3,000–5,000 ms, the next arithmetic triple, accompanied by a sound distractor, was presented and so forth.

Math problems (total of 162 different problems) were presented centrally on a 23-inch CRT monitor (Sony Trinitron Multiscan 200), set at a resolution of 1,680 × 1,050 with a refresh rate of 60 frames per second (i.e., 16.66 ms refresh interval). Three math problems were presented during each trial, as small vignettes, with black letters on a gray background (see Figure 1), spanning a visual angle of 6.9°, each for a duration of 2000 ms. The vignettes (math-problem boxes as shown in Figure 1) flickered on (50 ms) and off (50 ms) against a black background at a rate of 10 Hz to drive ssVEPs. Several authors have raised concerns regarding the use of ssVEP driving frequencies in the alpha-frequency range for ssVEP studies of attention (Ding et al., 2006). The resulting research has demonstrated that measures of the ssVEP that emphasize inter-trial phase consistency (e.g., averaging over large numbers of trials, inter-trial phase locking) at the driving frequency are sensitive to attention effects when using alpha-band driving frequencies (Keitel et al., 2010; Gulbinaite et al., 2019), which also tend to provide favorable signal-to-noise (Herrmann, 2001; Gruss et al., 2012), while being readily and regularly attained using most CRT or 3D-LED monitors (Wang and Nikolić, 2011).

One goal of the present study was to establish ssVEPs with a task used in an academic context rather than a laboratory task used in cognitive neuroscience. To this end, math problems were selected from the Woodcock-Johnson III Tests of Achievement – Math Fluency Subtest (Woodcock et al., 2001) and were simple arithmetic operations (addition, subtraction, and multiplication) of two numbers, with each number smaller than 10. Correct calculation results comprised only integer numbers in the single or double digits. Distractors were sounds from the International Affective Digitized Sounds (IADS, Bradley and Lang, 2000) and pink noise (with a frequency spectrum that followed a 1/f function) generated in MATLAB® using in-house software. The pink noise was included to serve as an added control, having no discernible content and no amplitude envelope modulation across the duration of its presentation (Wetzel et al., 2016). The duration of each sound stimulus was 6,000 ms, and it was presented simultaneously to the math problems (three problems at 2000 ms each). Naturalistic IADS stimuli were either high (20 sounds) or low (20 sounds) in terms of emotional arousal derived from normative ratings. High-arousing sounds were selected to be rated as maximally engaging, irrespective of pleasure, resulting in a total of nine judged as pleasant and 11 judged as unpleasant. Similarly, low-arousing stimuli were selected to be at the bottom of the normative arousal ratings, again regardless of sound pleasure, and comparable in content with the high-arousing stimuli. Paralleling previous work, the low-arousing stimuli tended to be perceived as moderately pleasant. Based on a nine-level pictorial scale (The Self-Assessment Manikin, SAM; Bradley and Lang, 1994), normative pleasure (1 = highly unpleasant to 9 = highly pleasant) and arousal evaluations (1 = low arousal to 9 = high arousal) were 5.93 (pleasure rating) and 3.91 (arousal rating) for low-arousing sounds (brush teeth, yawn, cows, Bach music, etc.). Corresponding figures for high-arousing sounds (female’s scream, attack, baby cry, Rock and Roll music, etc.) amounted to 4.51 (pleasure rating) and 7.08 (arousal rating), respectively^1^.

All auditory distractors (IADS stimuli and pink noise) were physically normalized to have the same root-mean squares (RMS) value of the digitally sampled sound waveform in an attempt to standardize the acoustic energy conveyed across conditions. This procedure resulted in a presentation intensity of 72 dB. Each sound was delivered twice, free-field *via* speakers to the left and right of the participant, for a total of 40 trials per distractor condition. For the first presentation of each distractor, the order was freely randomized. After all stimuli were presented, the order for the second presentation was freely randomized again, such that repetition occurred only after each sound was delivered once. Fixation on the math problems was facilitated by showing a white dot at the beginning of each trial. The fixation period matched the inter-trial interval, which randomly varied (rectangular distribution) between 3,000 and 5,000 ms. At the end of each trial, participants were instructed to report the outcome of the three math problems in the correct order by typing the results on the number pad of a standard keyboard. Prior to the experiment, all participants performed five practice trials to become familiar with the stimulation and arithmetic task. Following the experiment, participants rated a subset of five high-arousing and five low-arousing sounds, randomly extracted from the entire distractor pool, as well as the pink noise stimulus on the dimensions of pleasure and arousal, using a paper and pencil version of the SAM.

### Electrophysiological Assessment

#### Data Acquisition

Electrophysiological data were collected from the participants’ scalp using an Electrical Geodesic system 257-sensor net (EGI, Eugene, OR). Electrodes covered the entire scalp and the lateral aspects of the face. Placement was based on measurements of head size and fiducials (Cz, left and right pre-auricular points, nasion, and inion), and the net was adjusted for these locations to match with anatomical landmarks of the participant. Scalp impedance was kept below 60 kΩ, as recommended for this high (200 mΩ) input impedance amplifier. The EEG was recorded at a sampling rate of 250 Hz at 16-bit resolution. A hardware elliptical bandpass filter was applied online between 0.1 and 90 Hz. The vertex electrode (Cz) served as the recording reference. Further processing and filtering were conducted offline.

#### Data Reduction

Continuous data were low-pass filtered at a frequency (3-dB point) of 40 Hz (12th-order Butterworth filter) prior to segmenting. Single epochs of 7,000 ms in length, encompassing 600 ms pre- and 6,400 ms post-onset of the stream with math problems accompanied by auditory distractors, were then extracted from the EEG signal. We adopted the artifact rejection procedure proposed by Junghöfer et al. (2000). In this framework, trials and sensors with artifacts are identified based on the distribution of statistical parameters of EEG epochs (absolute value, standard deviation, and differences between subsequent sample points). Sensors contaminated with artifacts throughout were replaced by statistically weighted, spherical spline interpolated values. A maximum of 12 channels was set for interpolation. Bad trials (containing more than 12 contaminated channels) were excluded as well. After this step, an average of 32.5, 33.9, and 34.0 trials (*SD_overall_* = 4 trials) were retained for averaging in the low-arousing, high-arousing, and noise distractor condition, respectively.

#### Steady-State Visual Evoked Potential Analyses

Artifact-free epochs of the voltage data were averaged for each of the three auditory distractor conditions. The time-varying amplitude measured at the stimulation frequency of 10 Hz was then extracted by means of a Hilbert transformation of the time-domain averaged data using in-house MATLAB® scripts: First, data were filtered by means of an eighth-order Butterworth bandpass filter with a width of 0.5 Hz around the center frequency of 10 Hz (cut-offs defined as 3-dB points). Then, the time-varying amplitude was extracted as the modulus of the bandpass-filtered signal and the Hilbert-transformed analytic signal for each time point.

### Statistical Analyses

Statistical analyses were performed using STATISTICA, version 10 (StatSoft, Inc., 2011) and JASP software, version 09 (JASP team, 2018).

#### Affective Ratings of Sound Distractors

Mean pleasure and arousal values for pink noise and each subset of high- and low-arousing sounds per participant were determined and submitted to separate repeated measures analyses of variance (ANOVAs), with Distractor Type (3; low-arousing sounds, high-arousing sounds, pink noise) as the within-participant factor. Greenhouse-Geisser corrected degrees of freedom were applied in cases where sphericity was violated and indicated as *p_GGcorr_*. Partial eta squared ($\eta_{p}^{2}$) values are reported as a measure of effect size (Cohen, 1988). Bonferroni-Holm corrected *t*-tests (*p_bonf-holm_*) were used to further investigate significant main effects (*p* < 0.05).

#### Statistical Inference: Measures of Emotional Distraction

Statistical analyses took a two-pronged approach to seek convergent validity of frequentist and Bayesian statistical models. First, repeated measures ANOVA was conducted on both dependent variables, namely error rate and ssVEP amplitude (for details, see below), with an alpha-level set to 0.05. Greenhouse-Geisser corrected *p*-values were provided in cases where the assumption of sphericity was violated and effect sizes (i.e., $\eta_{p}^{2}$) are reported. Second, we calculated Bayes factors for each model of interest, compared to the null model of no effects based on main effects of Distractor Type (for error rate and ssVEP amplitude), as well as main effects of Time Window, Distractor Type, and their interaction (only ssVEP amplitude; for details see below). Equal priors were assumed for each model included in the ANOVA design (i.e., 0.5 for error rate models, 0.2 for ssVEP amplitude models; note that these are not priors for making a correct response or for the ssVEP to occur, but for each linear model in the ANOVA design; see Results and Wagenmakers et al., 2018). Percentages of estimation error for the Bayes factors are given throughout.

##### Error Rate

The percentage of correctly completed math problems was calculated for each sound distractor type (low- and high-arousing stimuli, as well as pink noise) and participant. Math performance was expressed in proportion of errors, relative to the total number of trials per distractor condition and subsequently submitted to repeated measures ANOVA, with Distractor Type (3; low-arousing sounds, high-arousing sounds, pink noise) as the within-participant factor. Bonferroni-Holm corrected *t*-tests were applied to follow-up a significant main effect.

##### ssVEP Amplitude

**Figure 2:**
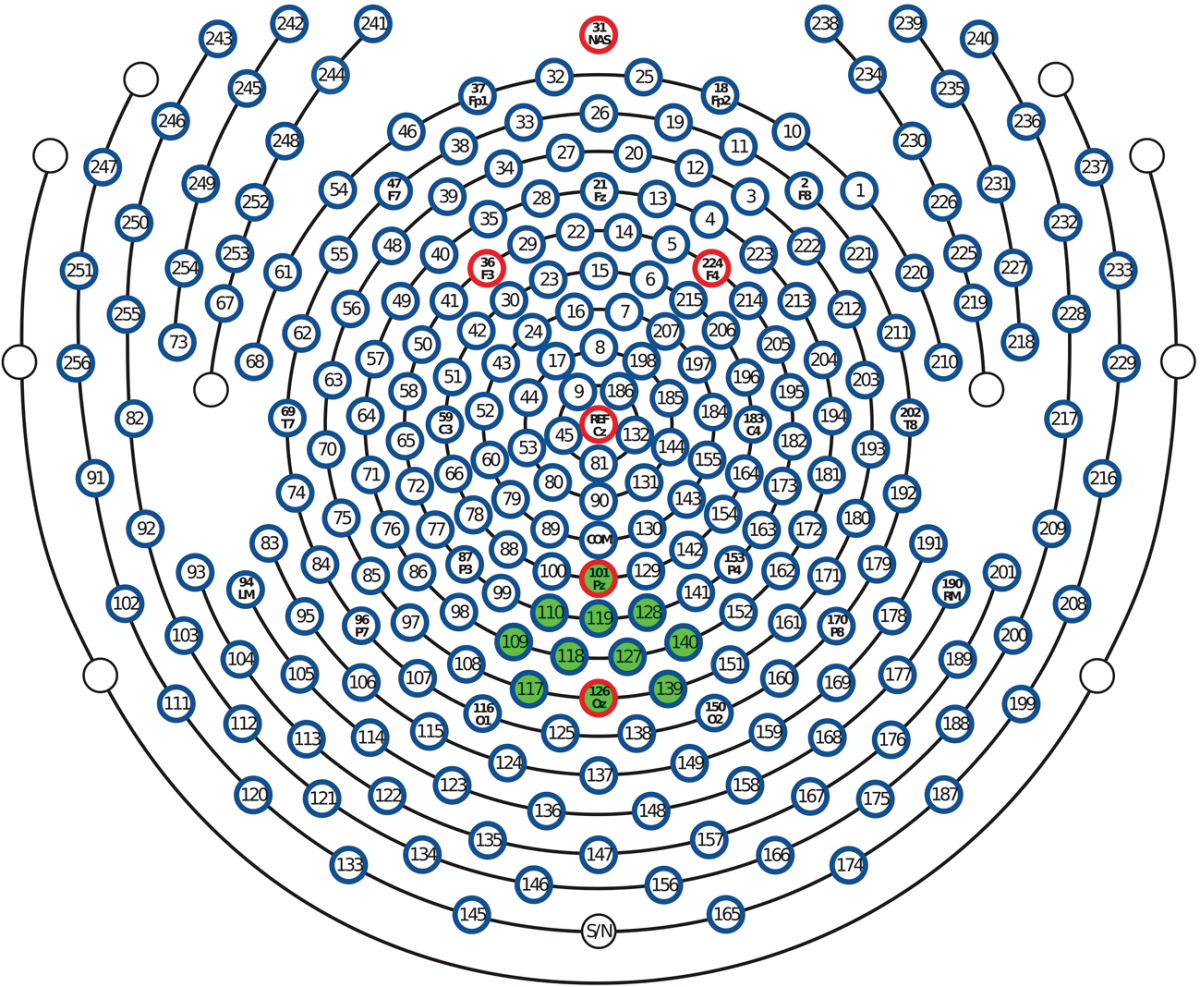
Layout of the 257-sensor array. Posterior electrodes are shown at the bottom of the figure. Green-shaded electrode sites, including Pz and Oz, were selected to form occipital regional means entering frequentist and Bayesian statistical models.

ssVEP amplitudes were averaged in an occipital cluster of 11 electrodes between Pz and Oz (see Figure 2) where the signal was most pronounced, resulting in occipital regional mean amplitudes. To evaluate modulation of the ssVEP signal across time, ssVEP amplitude was then averaged separately for two time windows (early: 400–2,600 ms and late: 3400–5,600 ms). These periods were chosen to avoid contamination of the ssVEP with the onset and offset event-related potential and to minimize overlap in the middle of the epoch due to the smearing of the time-frequency (i.e., Hilbert) transformation, which had a time domain uncertainty (full width at half maximum) of 129 ms. Amplitude means of the ssVEP signal were submitted to repeated measures ANOVA crossing the within-participant factors, Time Window (2; early, late) and Distractor Type (3; low-arousing sounds, high-arousing sounds, pink noise). Post-hoc inspection of significant results was effected by Bonferroni-Holm corrected *t*-tests.

## Results

### Post-Experimental Affective Ratings of Sound Distractors

Using the nine-point SAM scale, participants’ ratings on the arousal dimension showed a systematic variation by Distractor Type, *F*(2, 38) = 5.76, *p_GGcorr_* < 0.011, $\eta_{p}^{2}$ = 0.23. Bonferroni-Holm corrected post-hoc tests of the significant ANOVA result indicated that high-arousing sound distractors (mean ± SEM; 4.65 ± 0.59) were rated as more arousing than low-arousing exemplars (2.90 ± 0.37) and pink noise (3.15 ± 0.39), *t*(19) = −3.14, *p_bonf-holm_* = 0.010 and *t*(19) = 2.69, *p_bonf-holm_* = 0.021, respectively. Arousal judgments for pink noise and low-arousing distractors did not deviate significantly from each other, *t*(19) = −0.45, *p_bonf-holm_* = 0.656. In terms of the pleasure dimension, students’ judgments did not vary as a function of distractor content (low-arousing: 4.75 ± 0.38; high-arousing: 5.05 ± 0.36; pink noise: 5.15 ± 0.42, *F*(2, 38) = 0.31, *p_GGcorr_* < 0.702, $\eta_{p}^{2}$ = 0.02).

### Performance in the Intermodal Distraction Task

**Figure 3:**
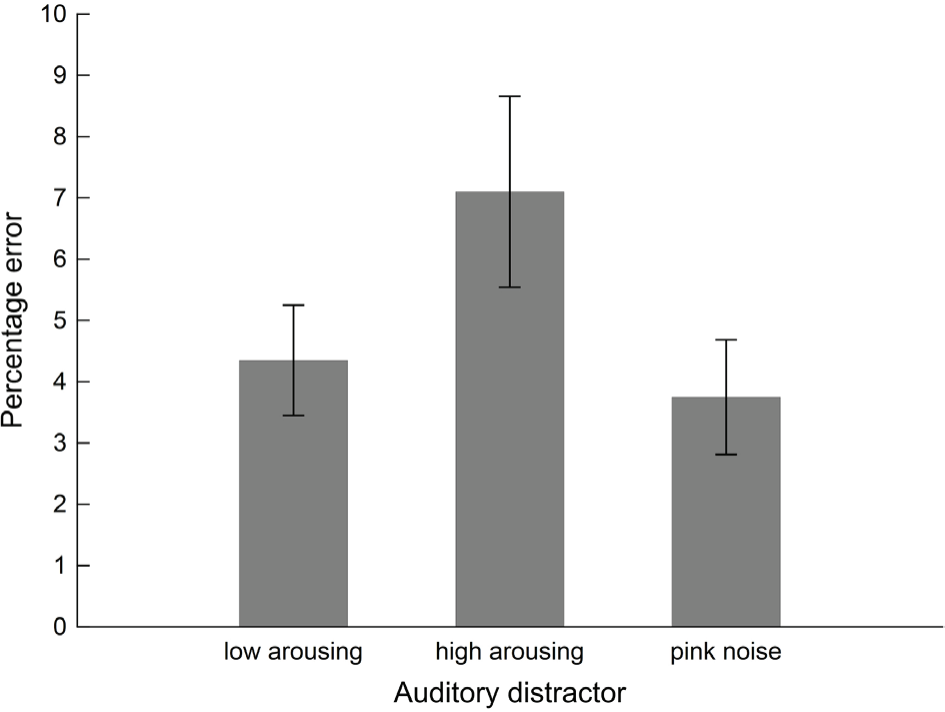
Performance of the students sample (*n* = 20) in the intermodal distraction task. Mean values of error percentages during math calculations are shown as a function of distractor sound. Vertical bars indicate standard errors of mean. The presence of high-arousing sound distractors impaired calculation performance (i.e., higher error percentage) compared to both low-arousing and pink noise exemplars.

Interference exerted by the sound distractors on performance in the arithmetic task varied with Distractor Type, *F*(2, 38) = 4.58, *p_GGcorr_* < 0.023, $\eta_{p}^{2}$ = 0.19, as illustrated in Figure 3. Bonferroni-Holm corrected post-hoc tests showed significantly diminished performance (i.e., higher error rate) in the presence of high-arousing sound distractors (0.07 ± 0.02), compared to both low-arousing stimuli (0.04 ± 0.01), *t*(19) = −2.33, *p_bonf-holm_* = 0.050, and pink noise distractors (0.04 ± 0.01), *t*(19) = 2.84, *p_bonf-holm_* = 0.022, during which participants performed better. Bayesian analysis converged with the frequentist ANOVA finding: The Bayes factor for the model in which accuracy varied with content was 3.35 (estimation error = 0.66%). This factor reflects that the data were 3.35 times more likely to arise from variations in content than from a null model in which Distractor Type did not have an effect and as such provides substantial evidence for the former (Distractor Type) model.

### Steady-State Visual Evoked Potentials Evoked by the Math-Problem Stream

**Figure 4:**
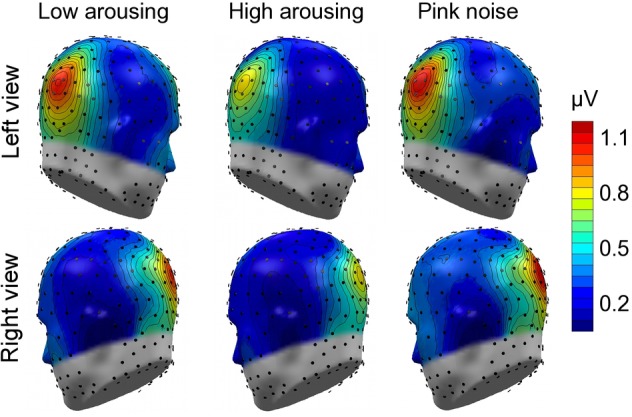
Topographical maps: Cortical response to the math-problem triplets. Left- and right-hemisphere views of grand mean ssVEP-amplitude distribution across the scalp (spline-interpolated maps) for the study group of 20 participants. The illustration represents the mean energy across the early and late time segments (400–2,600 ms and 3,400–5,600 ms, respectively), selected to extract the ssVEP signal. Prominent ssVEP amplitudes at the expected frequency of 10 Hz occurred at occipital sites across all auditory distractor conditions.

**Figure 5:**
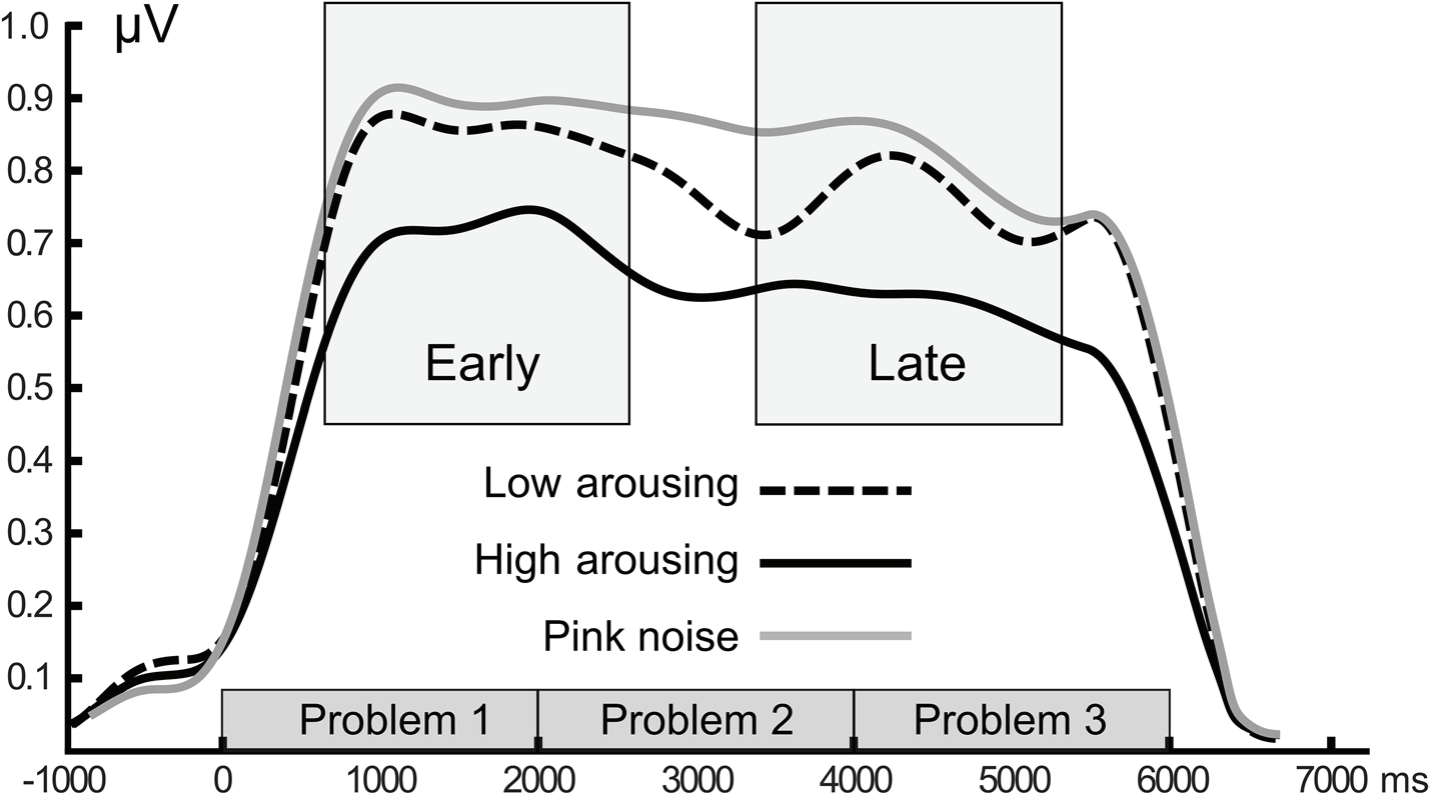
Grand mean time-varying ssVEP amplitude. Time course of the ssVEP amplitude over occipital sensors evoked by the math-problem stream for the three sound distractor conditions. Grand means of 20 participants are depicted. Time windows used for statistical analysis are shown as gray boxes. Amplitude shows enhanced distraction (i.e., lower signal strength) when high-arousing sounds were presented.

**Figure 6:**
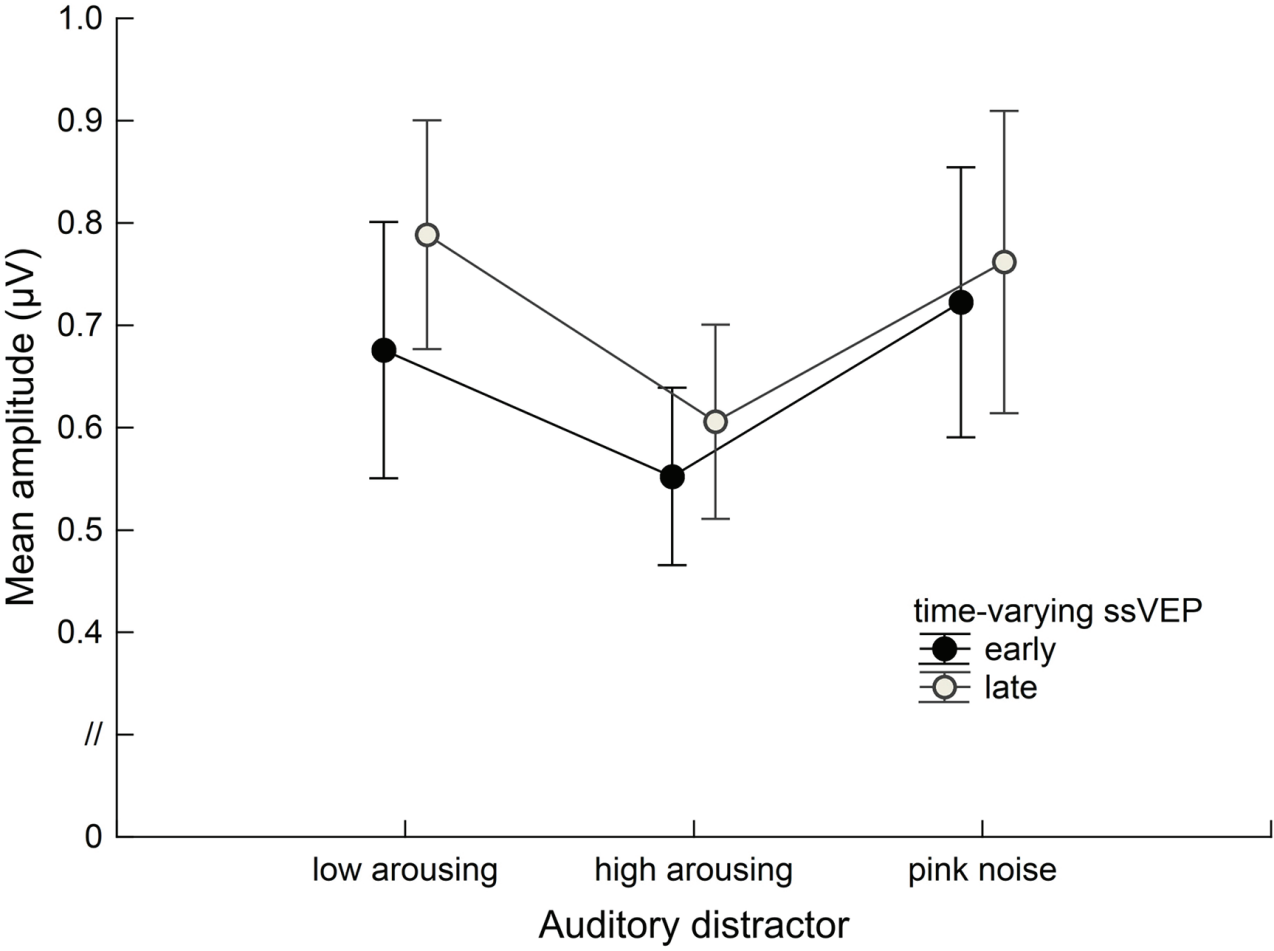
Group data (*n* = 20) for the time-varying ssVEP signal in the early and late time ranges. Mean amplitudes to the math-problem stream are shown as a function of auditory distractor type during early (400–2,600 ms) and late (3400–5,600 ms) time windows. Vertical bars indicate standard errors of mean. The presence of high-arousing sounds during math calculations was associated with diminished cortical responses compared to both low-arousing stimuli and pink noise. This distraction occurred across the time ranges of interest.

Math problems reliably evoked steady-state responses at the expected frequency of 10 Hz, with the greatest overall ssVEP amplitudes across all sound distractor conditions occurring at midline posterior sensors (see Figure 4 for the topographical distribution of ssVEP amplitude). The grand mean time-varying energy of the signal over occipital sensors as quantified by the Hilbert transform is shown in Figure 5. We compared the three distractor conditions during early and late segments of math-problem viewing in a repeated measures ANOVA with factors of Time Window and Distractor Type (for cell descriptives, see Table 1). While the effect of Time Window did not reach the 5%-significance level, *F*(1, 19) = 3.24, *p* < 0.088, $\eta_{p}^{2}$ = 0.15, a main effect of Distractor Type occurred, *F*(2, 38) = 4.49, *p_GGcorr_* < 0.029, $\eta_{p}^{2}$ = 0.19, which indicated that the ssVEP varied with the sound distractors. Bonferroni-Holm corrected *t*-tests characterized this difference as related to the emotional arousal of the distractor, with high-arousing distractor sounds prompting diminished ssVEP amplitude to the math problems, compared to both low-arousing, *t*(19) = 2.51, *p_bonf-holm_* = 0.033, and pink noise, *t*(19) = −2.67, *p_bonf-holm_* = 0.033, distractors. Notably, this effect did not interact with the factor of Time Window (early vs. late), *F*(2, 38) = 1.17, *p_GGcorr_* < 0.311, $\eta_{p}^{2}$ = 0.06, suggesting the presence of the intermodal distractor effect throughout the math-problem viewing epoch (see Figure 6). The same conclusion was also supported by the Bayesian analysis in which the data were 32.82 times more likely to arise from a distractor main effect model compared to the null model (estimation error = 0.75%).

**Table 1:** Cell descriptives of the steady-state response by time window and distractor type for the participant group (*n* = 20).

	Sound distractor		
	Low arousing	High arousing	Pink noise
ssVEP segment			
Early	0.68 ± 0.13	0.55 ± 0.09	0.72 ± 0.13
Late	0.79 ± 0.11	0.61 ± 0.09	0.76 ± 0.15

Means ± standard error of the means are shown.

## Discussion

The current study sought to investigate the extent to which the presence of sound distractors varying in emotional intensity affects the behavioral performance and visuocortical response in a primary task involving simple arithmetic operations. To obtain robust and readily identifiable visuocortical signals during intermodal distraction, single-digit math problems (addition, subtraction, and multiplication) were flickered on and off at a temporal rate of 10/sec, eliciting ssVEPs, separable in the time-frequency domain from auditory responses and ongoing noise. We found that the presence of high-arousing sounds, such as a crying baby and Rock and Roll music, during the arithmetic task prompted diminished accuracy and reduced visuocortical ssVEPs, compared to trials in which low-arousing (e.g., yawn, Bach music) or pink noise distractors were presented to the auditory modality.

The pattern of visuocortical amplitude modulation reported here is consistent with studies using visual distractors during ssVEP primary tasks (e.g., Deweese et al., 2014). In these studies, a reduction in ssVEP amplitude to the task-relevant stream is typically observed while viewing arousing distractor pictures in the background (e.g., Müller et al., 2008; Hindi Attar et al., 2010; Hindi Attar and Müller, 2012). Such interference by emotionally arousing picture distractors tends to be relatively early but brief, in that distraction effects typically extend from around 500 to 1,500 ms following the onset of the distractor, followed by a period in which such effects are absent. By contrast, using the high temporal fidelity of electrophysiological data, we found that auditory distraction effects on visuocortical processing were temporally sustained throughout the epoch. This temporal pattern is consistent with research that demonstrated persistent visual emotional distraction when observers were selected to exhibit strong engagement by a specific distractor category, for instance, participants high in social anxiety (Wieser et al., 2012) or high in snake fear (Deweese et al., 2014). Because the interference effects exerted by low-arousing sounds and pink noise did not differ, it is unlikely that the temporally sustained distraction effects observed here solely reflect the rich temporal structure of naturalistic auditory stimuli, which keep conveying information as time passes. Instead, it appears that the conveying of motivationally relevant auditory information is a necessary condition for distraction effects to occur. Given the known problems associated with using naturalistic stimuli in terms of controlling physical stimulus properties, future research may systematically vary these properties to characterize the impact of emotional content versus physical properties of naturalistic stimuli.

Research designs that allow researchers to quantify emotional distraction effects on concurrent visual tasks possess ecological validity, because they closely resemble real-life learning situations. Users of learning software or users researching for content on the World Wide Web often encounter distractors such as pop-up windows. The ability to re-engage attention to a task at hand is also important in the classroom, when interacting with course material that contains, for instance, engaging cartoons, alternating with intrinsically less interesting equations or learning content. The current study complements and converges with earlier work that investigated this problem using behavioral data. For example, Heim et al. (2013b) implemented an emotional distraction task developed previously for studies with adults (Ihssen et al., 2007) to examine distraction dynamics in early adolescents. Specifically, Heim and collaborators asked 11- to 13-year-olds to perform a lexical decision about letter strings (the targets) shortly after being briefly presented with task-irrelevant pictures. They found that both pleasant and unpleasant pictures impaired processing of subsequent word targets, measured as lexical decision time delays of approximately 50 ms, compared to neutral photographs. These emotional distraction effects were reliably observed across different time intervals between distractors and targets, including when the target was presented 600 ms after the offset of an emotional distractor. Thus, emotional stimuli capture and hold shared cognitive resources, leading to depletion of limited capacity over time and ultimately preventing optimal processing the target event.

In sum, the present findings support the distraction hypothesis, pointing towards strong intermodal distraction costs, exerted by emotionally engaging task-irrelevant sounds on concurrent visual processing. This interpretation is in line with event-related potential and functional neuroimaging work of intermodal interactions between emotional and task cues (e.g., Keil et al., 2007; Domínguez-Borràs et al., 2017). For example, evolutionary old motive circuits such as those centered around the amygdaloid bodies may amplify the neural response to emotionally engaging stimuli (Sabatinelli et al., 2009), whereas visuocortical amplification based on task may reflect bias signals originating in frontal cortex (Barceló et al., 2000). Thus, future work may want to examine competition at the level of brain networks, using the ssVEP signal as a reference point for detecting and quantifying neural communication during emotional distraction. The finding that both low-level visuocortical (luminance) processing and math performance were affected by auditory distraction may point to broader implications for educational neuroscience and education science in general. For example, recent advances in the neuroscience of language and number processing have identified the importance of widespread networks for academic ability. These networks are thought to build on evolutionarily older circuits that mediate functions such as spatial processing, object/shape processing, and intermodal integration (Dehaene, 2013). Here, we raise the possibility that these networks in turn interact with the neural machinery underlying the neurocomputation of motivational value, which affects the allocation of attentional resources. Thus, the present findings highlight the role of basic motivational processes in the translation of academic cognition into action.

## Data Availability

The datasets generated for this study are available on request to the corresponding author.

## Author Contributions

SH and AK designed the study protocol and equally contributed to the interpretation of results and writing of manuscript. SH conducted statistical analyses. AK conducted electrophysiological analyses.

### Conflict of Interest Statement

The authors declare that the research was conducted in the absence of any commercial or financial relationships that could be construed as a potential conflict of interest.

## Footnotes

^1^IADS stimuli (numbers are given) were as follows: low-arousing sounds—113, 132, 151, 206, 225, 251, 262, 270, 400, 602, 700, 701, 704, 707, 720, 725, 726, 811, 812, 816; high-arousing sounds—106, 110, 200, 220, 261, 277, 279, 291, 310, 352, 360, 415, 420, 422, 424, 502, 712, 730, 815, 820.
